# A Comparative Study of Impedance *versus* Optical Label-Free Systems Relative to Labelled Assays in a Predominantly Gi Coupled GPCR (C5aR) Signalling

**DOI:** 10.3390/bios2030273

**Published:** 2012-07-26

**Authors:** Reena Halai, Daniel E. Croker, Jacky Y. Suen, David P. Fairlie, Matthew A. Cooper

**Affiliations:** Institute for Molecular Bioscience, The University of Queensland, Brisbane, QLD 4072, Australia; E-Mails: r.halai@uq.edu.au (R.H.); d.croker@uq.edu.au (D.E.C.); j.suen@uq.edu.au (J.Y.S.); d.fairlie@uq.edu.au (D.P.F.)

**Keywords:** GPCR, impedance and optical biosensors, label-free, secondary messenger, signal transduction

## Abstract

Profiling ligand function on G-protein coupled receptors (GPCRs) typically involves using transfected cells over-expressing a target of interest, a labelled ligand, and intracellular secondary messenger reporters. In contrast, label-free assays are sensitive enough to allow detection in native cells, which may provide a more physiologically relevant readout. Here, we compare four agonists (native agonists, a peptide full agonist and a peptide partial agonist) that stimulate the human inflammatory GPCR C5aR. The receptor was challenged when present in human monocyte-derived macrophages (HMDM) *versus* stably transfected human C5aR-CHO cells. Receptor activation was compared on label-free optical and impedance biosensors and contrasted with results from two traditional reporter assays. The rank order of potencies observed across label-free and pathway specific assays was similar. However, label-free read outs gave consistently lower potency values in both native and transfected cells. Relative to pathway-specific assays, these technologies measure whole-cell responses that may encompass multiple signalling events, including down-regulatory events, which may explain the potency discrepancies observed. These observations have important implications for screening compound libraries against GPCR targets and for selecting drug candidates for *in vivo* assays.

## 1. Introduction

G-protein coupled receptors (GPCRs) comprise 25–30% of all targets for marketed pharmaceuticals [1,2]. Discovery of drug candidates against GPCRs has typically involved the use of (i) transfected cell lines over-expressing the target receptor; (ii) a labelled ligand for measuring competitive binding affinity for the GPCR; and (iii) one or two selected secondary messengers (e.g., ERK1/2, cAMP, Ca^2+^) for assessing G-protein dependent intracellular function [3,4,5,6]. Decisions to profile *in vivo* activities have thus tended to be based on functional characterisation of a compound as an agonist or antagonist in just one or two pathway-dependent assays, with the outcome potentially compromised by unpredicted functional activities in other pathways. Reporter tags or labels, which do not interfere with the biology, are required to measure downstream secondary messengers. Non-invasive assays that use native cells, unlabelled ligands and reporters, may instead provide a more holistic and physiologically relevant assessment of GPCR function. 

**Figure 1:**
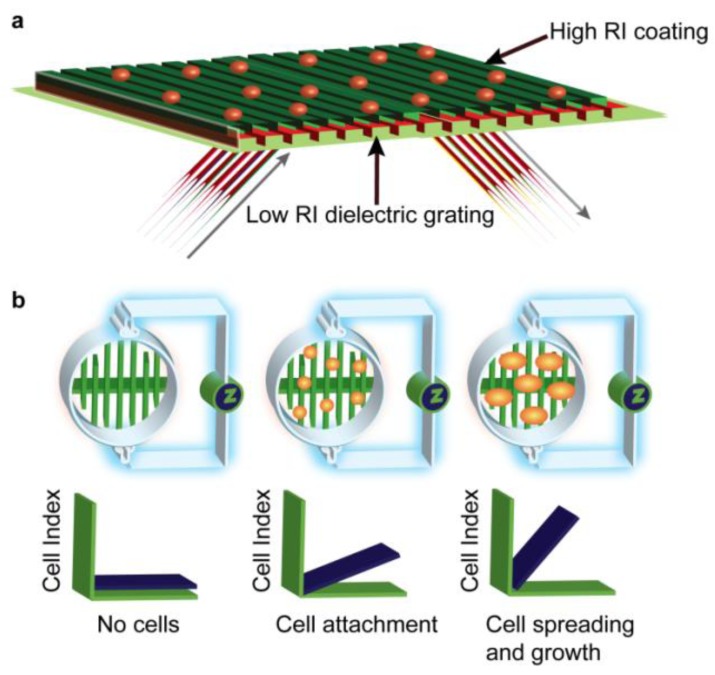
Overview of optical resonant waveguide and cell impedance label-free platforms. (**a**) Cross-section of the resonant waveguide grating optical biosensors in each well of a 384-well plate. A coating with a high index of refraction on the sensor surface reflects only a narrow band of wavelengths when illuminated with an optical beam. The incident angle of the reflected beam is sensitive to mass redistribution within the cell up to ~150 nm from the surface [17]; (**b**) Cells are plated onto gold microelectrode arrays, which when stimulated with a low voltage; generate an electric field sensitive to changes in the properties of a cell. Impedance measurement in Cell Index (CI) is zero when cells are not present. The impedance increases as cells attach and spread across the electrodes [19].

The physical basis and general capabilities of commercially available label-free platforms have been reviewed previously [7,8,9,10,11]. Two generally adopted label-free detection platforms based on optical and impedance transduction are highlighted in Figure 1 [12]. However, there are other label-free transductions modes; acoustic biosensors such as the Quartz Crystal Microbalance in which acoustic frequency shifts are influenced by mass and viscoelastic property changes of receptor-analyte interactions, Isothermal Titration Calorimetry, which measures changes in heat as a result of binding complex formation, as well as many others [13]. Furthermore, there are also various forms of optical biosensors, such as, surface plasmon resonance (SPR) or resonant waveguide grating (RWG). This study explores the Corning EPIC^®^ system which is an optical biosensor based on resonant waveguide grating (RWG) [14,15]. Alternatively referred to as a “photonic crystal”, it is comprised of a periodic arrangement of dielectric material in two or three dimensions. If the periodicity and symmetry of the crystal and dielectric constants of the materials are chosen appropriately, the photonic crystal will selectively couple energy at specific wavelengths (Figure 1). When embossed at the bottom of a 96- or 384-well plate, the crystal structure geometry can be designed to concentrate light into extremely small volumes, so that the sensor is sensitive to “mass changes” in cells close (~150–200 nm) to the base of the well plate [16,17]. 

Impedance biosensors fall under the umbrella of “electrical biosensors”, which encompass impedance, voltametric and amperometric/coulometric sensors, where the latter two utilise an electrode to measure current as a function of applied voltage [18]. The xCELLigence system from ACEA/Roche [19] used in this study utilises impedance measurements to quantify the cellular response of adherent cells [20,21,22]. Inter-digitated gold microelectrodes line the base of a 96- or 384-well plate to which cells attach. The inter-digitated co-planar electrodes of the system form a capacitor, an alternating voltage is applied at a range of frequencies V(f), and the resulting electrical currents I(f) are measured. Impedance is a function of frequency Z(f) = V(f)/I(f). At lower frequencies the applied voltage induces an extracellular current, while at higher frequencies the voltage passes through the cell membrane [23]. Cells and electrodes form an electrical circuit coupled to an impedance analyser (Figure 1(b)). When cells are exposed to ligands that cause signal transduction, cellular changes interfere with induced extra- and trans-cellular current, thereby impacting on impedance. Output data analysis gives quantitative measures of cell activation, ligand-receptor specificity, and agonist/antagonist potency. How ligand-induced signals couple to platform responses for both instruments is not yet fully understood, but concentration-dependent ligand responses do correlate with activation of the receptor and downstream signalling, in particular cytoskeletal rearrangement [24,25].

Here, we use a GPCR of the innate immune system, C5aR (CD88), to report assay data comparing phenotypic readouts of receptor activation from optical resonance waveguide grating and electrical cell impedance, and correlate these back to traditional secondary messenger reporter assays. The human C5a receptor (C5aR) is a potent chemoattractant and pro-inflammatory mediator in the immune response [26]. It is also implicated in many inflammatory disorders, including sepsis [27], chronic lung disease [28] and atherosclerotic lesions [29]. It is expressed in myeloid cells such as neutrophils [30] and macrophages [31], as well as many non-myeloid cells [32]. Its endogenous ligand is a 74 residue protein known as C5a, which is rapidly degraded to C5a des-Arg (minus its C-terminal arginine) by carboxypeptidases [33]. Although signalling pathways have been extensively mapped for C5aR in many different cell types [26], here we use it as a tool to probe and identify similarities and differences between optical and impedance label-free technologies. This report compares signalling induced in HMDM and human C5aR-transfected CHO cells by human C5a, C5a des-Arg, and two hexapeptide agonists. Results were comparable using the label-free impedance and optical biosensor, whereas there were notable differences in functional characterisation using secondary messenger assays. Responses in HMDM and CHO-C5aR cells were different, highlighting the importance of using intact native cells with endogenous receptor densities for ligand screening programs.

## 2. Experimental Section

The two label-free instruments (xCELLigence and EPIC^®^) analysed in this studied were employed because of their availability in our laboratory. To undertake these studies using these label-free instruments, the parameters were tailored to the requirements of the system analysed. Specific details of experimental design can be found in this section, with further discussion on chosen parameters in the “Results and Discussion” section.

### 2.1. Ligands

C5a (non-glycosylated) was purchased from Sigma Aldrich and Sino Biological Inc, and C5a des-Arg (glycosylated) from Merck. A peptide full agonist FKPChaChaR = C028 (*NMePhe-Lys-Pro-dCha-dCha-dArg-COOH*) [34] and a peptide partial agonist FKPChaNalR = C061 (*NMePhe-Lys-Pro-dCha-dNal-dArg-COOH*) [34] were synthesized in-house and are referred to as either peptide agonist for FKPChaChaR = C028 or peptide partial agonist for FKPChaNalR = C061. Note *Cha* *=* cyclohexylalanine and *Nal* = I-naphthyl.

### 2.2. Cell Culture

Human monocytes were isolated from blood donations to the Australian Red Cross Blood Service (Kelvin Grove, Queensland). The buffy coat and the peripheral blood mononuclear cell layer were separated using density gradient centrifugation with sterile Ficoll-Paque Plus (GE Healthcare). CD14 selection was used to isolate CD14+ cells. MACS magnetic beads (Miltenyi Biotec) were incubated with the cells for 15 min at 4 °C. Cells were passed through a LS column (Miltenyi Biotec) to select for the CD14+ cells, as stated in the manufactures instructions. Selected monocytes were plated in a square sterilin petri dish at a density of 1.5 × 10^7^ in the presence of Iscove’s Modified Dulbecco’s Medium (IMDM) (Invitrogen Life Technologies) containing L-glutamine supplemented with 10% Fetal Bovine Serum (FBS), 50 IU/mL penicillin and 50 μg/mL streptomycin. The cells were differentiated over 7 days at 37 °C and 5% CO_2_ to human monocyte-derived macrophages (HMDM) using human macrophage colony-stimulating factor (M-CSF) (Peprotech) [35]. 

CHO cells transfected with human C5aR (Perkin Elmer) were cultured in a buffer of Ham’s F12, 10% FBS, 100 IU/mL penicillin, 100 μg/mL streptomycin and 400 μg/mL G418 (Invitrogen Life Technologies). The CHO-K1 cells (Cellbank Australia) were maintained in Ham’s F12, 10% FBS, 100 IU/mL penicillin and 100 μg/mL streptomycin. Cells were passaged at 80% confluence using 0.05% trypsin: EDTA in phosphate-buffered saline (PBS), pH 7.4. All cells in culture, once seeded, were incubated at 37 °C with 5% CO_2_, unless stated otherwise.

### 2.3. Membrane Preparation

Membranes were prepared from CHO-C5aR cells by modification of a previously described method [36]. Briefly, cells were washed with PBS, pH 7.4, and harvested into buffer (50 mM Tris-HCl, pH 7.4, 5 mM MgCl_2_, 1 mM CaCl_2_) before homogenising with a Polytron homogeniser for 3 min on setting 22. Cells were centrifuged at 150× g for 5 min at 4 °C. The supernatant was recovered and centrifuged at 22,000× g for 1 h at 4 °C. Membrane pellets were then resuspended in 0.4 mL of ice-cold assay buffer A (50 mM Tris-HCl, pH 7.4, 5 mM MgCl_2_, 1 mM CaCl_2_, 0.5% bovine serum albumin (BSA) (Sigma Aldrich) containing 10% glycerol) with aliquots stored at −80 °C. The protein concentration of the membrane preparations was determined by the Bradford method [37].

### 2.4. Receptor Binding Studies

The scintillation proximity assay (SPA) was used to study ligand binding interactions with the receptor. The basic principle of the assay involved coupling receptor membrane preparations to coated SPA beads. When a radiolabelled ligand, which is able to bind to the receptor coupled onto the SPA bead, interacts with the receptor it will be close enough in proximity to activate the SPA bead. The radiolabel emits radiation that leads to the production of a light signal by activation of the scintillant in the bead [38]. More specifically for our studies, receptor binding studies were performed using Polyvinyltoluene (PVT) scintillation beads, which have wheat germ agglutinin (WGA) covalently attached on the surface (Perkin Elmer). These beads allow for the development of a homogeneous GPCR radioligand binding assay using cellular membranes or whole cells. Briefly, receptor SPA studies were performed in 96-well white isoplates with clear flat bottoms (Perkin Elmer). ^125^I-C5a (~25 pM) (specific activity, 2,200 Ci/mmol) (Perkin Elmer) was added to either CHO-C5aR membrane preparations (~2 µg/well) or HMDM (25,000 cells/well). SPA beads (200 µg/well) were then added, followed by the addition of various concentrations of competing ligands (6 pM to 100 µM) in a total volume of 80 µL of buffer A (CHO-C5aR) or B (HMDM) (50 mM Tris-HCl, pH 7.4, 0.5% BSA, and either 5 mM MgCl_2_, 1 mM CaCl_2_ (A) or 3 mM MgCl_2_, 0.1 mM CaCl_2_ (B)). The final reaction volume per well comprised 20 µL of ligand/buffer, 20 µL of PVT WGA SPA beads, 20 µL of membrane/cells and the assay was initiated by the addition of 20 µL of ^125^I-C5a. The plate was then sealed using TopSeal-A (Perkin Elmer) sealing film and incubated with shaking for 1 h at room temperature. Radioligand binding was then assessed for 30 s/well using a 1450 Microbeta scintillation counter (Perkin Elmer). All binding and subsequent assay data, unless stated otherwise, was analysed using GraphPad Prism 5.0c (GraphPAD Software Inc., San Diego, CA, USA). 

### 2.5. ERK Phosphorylation Assay

Alphascreen (Amplified Luminescent Proximity Homogeneous Assay Screen) SureFire phospho-ERK1/2 assay was performed according to manufacturers’ instructions (Perkin Elmer). AlphaScreen involves the use of two beads, denoted the donor and acceptor beads [39]. When both beads are bound as a pair to an analyte and the donor bead is excited, a singlet oxygen is released which activates the acceptor bead. As there is a distance constraint here, only beads in close proximity can lead to further reactions that produce the detectable chemiluminescent signal [39]. Briefly, HMDM and CHO-C5aR cells were serum starved and incubated in 96-well tissue culture plates (Nunc) overnight at 60,000 and 50,000 cells/well, respectively. Ligands were prepared in serum-free media at a final DMSO concentration of 1%. Ligands were incubated at room temperature for 10 and 15 min with HMDM and CHO-C5aR, respectively. Media was removed and cells were lysed with lysis buffer for 10 min on a shaker, 4 μL of lysate was transferred to a white 96-well half area plate (Perkin Elmer) and incubated in the presence of 7 μL of reaction mix. The plate was sealed with TopSeal-A, incubated for 2 h at 37 °C and measured on an Envision plate reader (Perkin Elmer). Each data point was performed in triplicate and repeated in at least three separate experiments. 

### 2.6. EPIC^®^ Label-Free Optical System (Corning)

**Figure 2:**
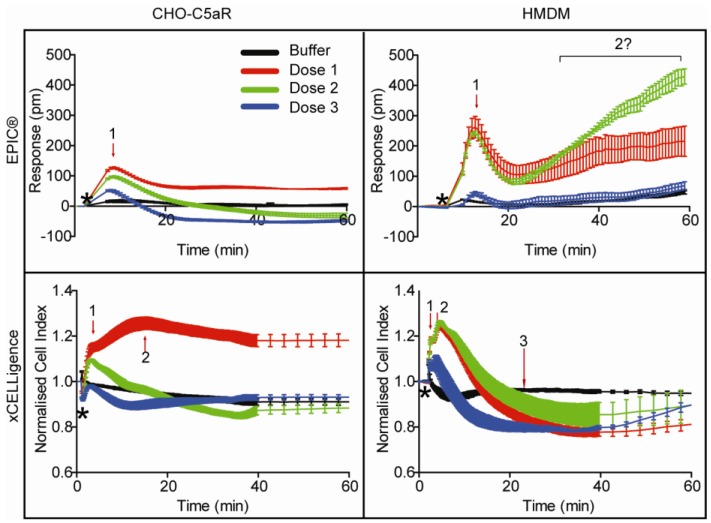
Three dose representative label-free profiles for C5a (red, blue and green) and baseline (black) in human monocyte-derived macrophages (HMDM) and CHO-C5aR cells on the EPIC^®^ and xCELLigence. The average of triplicates with SEM is shown. For CHO-C5aR cells, red is 250 nM, green is 83 nM and blue is 28 nM. For HMDM, red is 1 µM, green is 0.3 µM and blue is 0.1 µM. Arrows indicate peaks that may be indicative of different signalling pathways. Black star indicates point of compound addition. Question mark indicates ambiguous, but dose-dependent response.

HMDM were seeded, at 50,000 cells/well and CHO-C5aR and CHO-K1 at 7,500 cells/well, into fibronectin-coated cell-based EPIC^®^ plates (Corning) and incubated overnight. Prior to ligand addition, media was exchanged for HBSS (Invitrogen Life Technologies): 20 mM HEPES (Sigma Aldrich) buffer, supplemented with 0.5% DMSO, and allowed to equilibrate at 26 °C for 1 h (HMDM) or 2 h (CHO-C5aR and CHO-K1) within the instrument. Agonists were prepared at a final DMSO concentration of 0.5% in HBSS:HEPES buffer. After ligand addition, measurements were taken continuously for 1–2 h at 26 °C. Peak amplitude response (Figure 2) was measured for data analysis using Corning EPIC^®^ software. 

### 2.7. xCELLigence HT Label-Free Impedance System (Roche)

HMDM were seeded at 30,000 cells/well, CHO-C5aR at 5,000 cells/well and CHO-K1 at 7,500 cells/well and incubated overnight in 384-well E-Plates (Roche). Ligand preparation and cell treatment was as described for the EPIC^®^ protocol above. However, during the buffer exchange equilibration steps, cells were kept in the incubator at 37 °C with 5% CO_2_. HMDM were media exchanged with serum-free IMDM and incubated for 2 h prior to ligand addition with recordings at 37 °C. Ligands for the HMDM experiments were prepared in serum-free media. To ensure consistency with the EPIC^®^ data, time point for data analysis was determined by a number of different factors, mainly maximum response of predominant peak (Figure 2), but point at which the EC_50_ was stable over this time according to the RTCA software was also used to confirm the time point

## 3. Results and Discussion

### 3.1. Receptor Binding Assay

**Figure 3:**
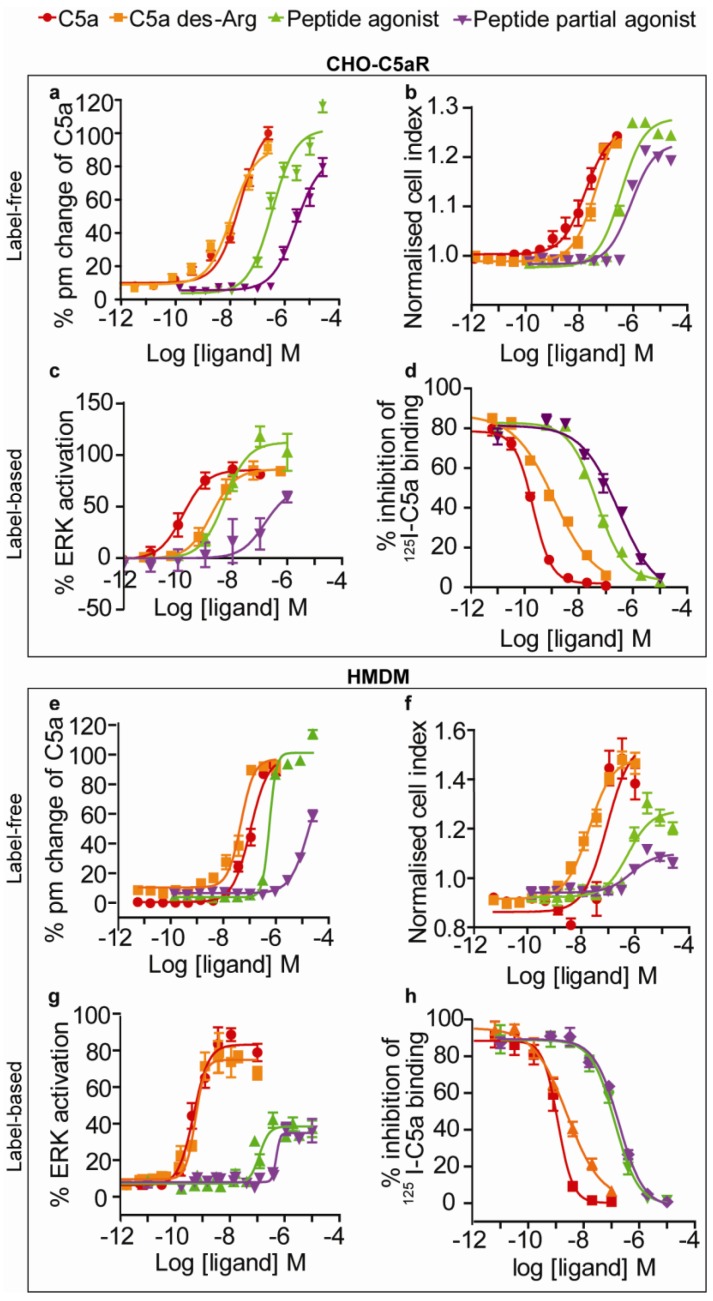
Label-free dose response curves on CHO-C5aR cells and HMDM. (**a**) Label-free response of all ligands on the EPIC^®^ optical system on CHO-C5aR cells (**b**) Label free response of all ligands on the xCELLigence impedance system on CHO-C5aR cells (**c**) Ligand response on Alphascreen secondary messenger ERK assay on CHO-C5aR cells and (**d**) I^125^ binding assay of all ligands on CHO-C5aR membranes (**e**) Label-free response of all ligands on the EPIC^®^ optical system on HMDM (**f**) Label-free response of all ligands on the xCELLigence impedance system on HMDM (**g**) Ligand response on Alphascreen secondary messenger ERK assay on HMDM and (**h**) I^125^ binding assay of all ligands on HMDM. Error bars represent standard error of the mean (n = 3 − 15).

There has been some inconsistency in the literature with regards to the activity of C5a to its reported “inactive” form, C5a des-Arg, in different cell types and across different assay platforms [30,33,40,41,42,43,44,45,46,47]. Most suggest a 10–1,000 fold reduction in potency for C5a des-Arg compared to C5a. It has also been shown that the de-glycosylated form of C5a des-Arg is 10-fold more potent than the native glycosylated form [41]. In HMDM the IC_50_ of C5a was 1.2 nM compared to 2.5 nM (~2-fold difference) for C5a des-Arg (Table 1). However, for CHO-C5aR cells, an IC_50_ of 0.2 nM and 1.2 nM (Table 2) (~6-fold difference) was observed for C5a and C5a des-Arg, respectively (Figure 3). The observed difference in binding affinity between the two cell types, may be due to the presence of other interacting partners/modulators in the HMDM that are absent in the CHO-C5aR cell line. Thus the binding affinities are dependent on the cellular background and can vary from one cell type to the other. The peptide partial agonist bound more tightly in HMDM relative to CHO-C5aR cells; however, this was not observed for any other ligand. The binding observed on the CHO-C5aR membrane was specific, since there was no specific binding to CHO-K1 membrane by ^125^I-C5a (data not shown).

**Table 1:** Potency of ligands (nM) on HMDM across the different platforms tested. Binding assay performed using 25 pM ^125^I-C5a. EC_50_/IC_50_ of compounds, with 95% confidence intervals (n = 3 − 8).

	Binding (IC_50_)	ERK (EC_50_)	EPIC^®^ (EC_50_)	xCELLigence (EC_50_)
**C5a**	1.2 (1–1.4)	0.47 (0.37–0.59)	107 (89–129)	89 (47–169)
**C5a des-Arg**	2.5 (1.9–3.4)	0.55 (0.42–0.72)	47 (38–56)	20 (15–27)
**Peptide agonist**	132 (111–157)	120 (86–167)	563 (526–603)	546 (293–1,016)
**Peptide partial agonist**	185 (141–242)	492 (227–1,068)	17380 (6,856–44,070)	709 (304–1,652)

**Table 2:** Potency of ligands (nM) on CHO-C5aR cells across the different platforms tested. Binding assay performed using 25 pM ^125^I-C5a. EC_50_/IC_50_ of compounds, with 95% confidence intervals (n = 3 − 16).

	Binding (IC_50_)	ERK (EC_50_)	EPIC^®^ (EC_50_)	xCELLigence (EC_50_)
**C5a**	0.2 (0.17–0.25)	0.15 (0.08–0.3)	24 (19–31)	16 (10–26)
**C5a des-Arg**	1.23 (0.78–1.95)	2 (1–3)	11 (9–15)	44 (34–57)
**Peptide agonist**	47 (36–60)	5 (2–11)	347 (262–461)	351 (292–421)
**Peptide partial agonist**	295 (147–594)	151 (16–1,382)	2626 (2,013–3,425)	819 (674–996)

### 3.2. ERK Phosphorylation Assay

Cells were serum starved overnight, as serum contributed to relatively high background counts. After serum starvation, cells were stimulated with agonists prepared in serum-free media. The EC_50_ for these ligands is highlighted in Table 1 and Table 2. In CHO-C5aR cells, the peptide agonist showed a potentiation of the response relative to the native ligand, whereas in HMDM the peptide agonist exhibited characteristics of a partial agonist. Despite the increased level of response observed for the peptide agonist in CHO-C5aR cells, the EC_50_ was still ~30-fold lower than that of C5a. At the highest concentration of the peptide partial agonist, only approximately half of the response relative to C5a was observed in both cell types. To confirm that these responses were specific to this receptor and not another endogenous receptor, these ligands were tested on CHO-K1 cells that had not been transfected (data not shown). No signal was observed for any ligand on CHO-K1 cells, verifying that these responses were C5aR specific. In both cell types, CHO-C5aR and HMDM, the rank order of ligand potencies was C5a > C5a des-Arg > peptide agonist > peptide partial agonist.

### 3.3. EPIC^®^ Label-Free Optical System

The optical based label-free EPIC^®^ system was used to assess the C5aR signalling profiles in our chosen cell types. Due to instrument configurations, where the temperature control unit was set at 26 °C, a few technical issues needed to be taken into consideration for our assays. The HMDM were sensitive in the EPIC^®^ platform to extended time in the HBSS assay buffer at the operating temperature of the instrument. Therefore, there were issues with baseline stability that were not observed for the CHO-C5aR cells (Figure 2). The HMDM were evaluated in serum-free IMDM on the EPIC^®^ system, but this failed to stabilise the baseline. The problem was overcome by incubating the HMDM in the instrument for a maximum of 1 h following buffer exchange with HBSS buffer. This improved the baseline stability for the assay and provided reproducible results. Immediate observations from CHO-C5aR *versus* HMDM EPIC^®^ profiles indicated greater complexity of signalling for the latter. A good signal, measured in picometers (pm), was observed on both cell types. The representative EPIC^®^ profile shown in Figure 2 for HMDM shows a greater response (pm) than in CHO-C5aR cells. However, the amplitude of this response varies in HMDM from donor to donor. Some donor HMDM show a near equivalence intensity in signal, whilst HMDM from other donors show greater or lesser signal responses. This is not surprising considering HMDM are primary cells isolated from human blood, where expression levels can vary from donor to donor. Due to these variations observed, the results were normalised to C5a. However, as expected, the signal responses from the CHO-C5aR cells are very consistent as all cells belong to one population. The overall signalling profiles on both cell types show an initial positive picometer response, which peaks ~5 min after compound addition and then decreases again. HMDM show an unexplained slight incline in the signal after the initial peak has declined, which, despite being dose dependent may just reflect an intrinsic drift in the cells with extended periods of time in a non-serum containing medium. The dose response curves for agonists on CHO-C5aR and HMDM using the EPIC^®^ are shown in Figure 3(a,e). These values were extrapolated from maximum peak responses, which occurred at ~5 min for both cell types, a time that concurs with proposed GPCR signalling kinetics [48]. The calculated ligand potencies were lower using the optical technology than those found using traditional secondary messenger reporter assays. However, the same rank order of ligand potencies was ascribed using the EPIC^®^ as secondary messenger assays in both cell types, with the exception of C5a des-Arg that was more potent than C5a in the EPIC^®^ assay.

### 3.4. xCELLigence Impedance Label-Free System

As this study aimed to compare optical with impedance measurements, all appropriate parameters used on the EPIC^®^ were replicated, where possible, on the xCELLigence. However, during optimisation there was a need to adjust some conditions to maintain healthy growth of the cells and to generate a stable signal. On the xCELLigence platform, the CHO-C5aR cells were subjected to the same procedure as that followed on the EPIC^®^ system described above, whereby cells underwent buffer exchange for 2 h prior to ligand addition, except that the incubation was performed in a 37 °C incubator with 5% CO_2_. However, for the HMDM, a lower cell density of 30,000 cells/well was optimal. For both cell types a reduced number of cells were required on the E-plates, relative to the EPIC^®^ plates. For this system, HMDM preferred and were assayed in serum-free media for 2 h over HBSS buffer for 1 h. A good signal, measured as cell index, was observed for both cell types. The signalling profiles generated using the xCELLigence indicated activation of additional signalling pathways in HMDM relative to CHO-C5aR (Figure 2). A few peaks which responded in a dose dependent manner were identifiable. The initial response observed on both cell types with this system, was faster than that observed in the EPIC^®^ profiles. Furthermore, distinct differences were observed in the dynamics of the profile, particularly in HMDM, which is discussed further in the discussion. The dose response curves for agonists on CHO-C5aR and HMDM using the xCELLigence are shown in Figure 3(b,f). For both cell types, these results were extrapolated from maximum peak response of the predominant peak, which occurred at ~3.5 min. The EC_50_ values observed for the agonists, relative to the secondary messenger assays, were higher; again indicating the potency ascribed to these compounds was lower when measuring a global response. However, the rank order potencies of these ligands was the same as that observed for the binding and the ERK assays, for both HMDM and the CHO-C5aR cells, with the exception of C5a des-Arg. 

### 3.5. Discussion

Agonist/antagonist affinity and function can be overestimated or biased by GPCR density, cell type and selection of the particular G-protein dependent pathway examined. Measurements of whole cell responses using optical and impedance technologies provide alternate methodologies for identifying compounds that bind to and modulate GPCRs, for subsequent Quantitative Structure-Activity Relationship (QSAR) studies, and for further elaboration towards drug candidates. The C5a receptor, which induces its inflammatory response by coupling predominantly to the Pertussis toxin sensitive Gαi2 [49,50], was chosen as a model receptor for analysis. The native ligand C5a, its predominant *in vivo* form C5a des-Arg, as well as full and peptide partial agonists reported previously [34], provided a useful tool set to investigate the suitability of these technologies for differentiating ligand potency and function. Ligand induced responses were analysed on a native cell type (HMDM) expressing the C5a receptor [51] *versus* a stably transfected CHO cell line. The parental non-transfected cell line was also analysed as a control to ensure the signals observed were specific to C5aR (data not shown). 

If a ligand is to induce a response, it must first bind to the receptor. The ligands were tested in a competitive ^125^I-C5a binding assay. All ligands were able to displace ^125^I-C5a, with both C5a and C5a des-Arg showing activity at low nanomolar concentrations. The IC_50_ for each of the ligands, with the exception of the peptide partial agonist, was higher on HMDM, indicating weaker binding relative to CHO-C5aR cells (Table 1 and Table 2). Upon confirmation of binding, the ligands were tested in an ERK1/2 phosphorylation assay because C5aR is known to predominantly couple to Gαi, which signals through this kinase [52,53]. In the ERK1/2 assay, with the exception of C5a des-Arg, all ligands were more potent on CHO-C5aR cells. Interestingly, the peptide agonist described as a “full agonist”, in a myeloperoxidase release assay in neutrophils at this receptor [34], exhibited partial agonist activity on HMDM. The lower binding affinity and potency of the ligands, for HMDM, in the ^125^I-C5a binding assay and ERK1/2 assay is not entirely surprising. GPCRs are known to exist in complex organisations, where their binding and functional activity can be influenced by surrounding interacting proteins. Studies in the literature have highlighted differences between transfected and natively expressed receptors, such as binding affinity differences [54] and differences in coupling to GTP-binding proteins [55]. Furthermore, it is now more widely accepted that GPCRs may form dimers/oligomers (homo or hetero), that can influence the pharmacological profile of the ligand (Well reviewed by Prinster *et al*. [56]). In our study it is possible that in HMDM the presence of C5L2, a known binding but non-signalling “scavenger” GPCR for C5a [57], is regulating the readily available pool of C5a and thereby reducing the binding/functional response in HMDM. As C5L2 has not been documented to be expressed in CHO cells, this may explain the differences seen between the transfected CHO-C5aR cells and the HMDM. It was previously thought that when a ligand binds, it induces an equal signalling response along all pathways associated with the receptor; this is now known not to be the case for most ligands. Traditionally, a single secondary messenger assay was used to rank ligands in GPCR screening, but the limitations of this approach are now being recognised. To decipher all signalling pathways using separate secondary messenger reporters, a ligand has to be independently tested in parallel in many different reporter assays. This is extremely time consuming and prohibitively expensive. A label-free platform, together with receptor-binding studies, has the advantages of being a faster, more efficient “first pass” screen for ligands, without being biased by the choice of reporter signal to be monitored. In this study, the impedance based xCELLigence system was used to measure C5aR activation. The cell index readout from this technology represents a change in the electrical impedance when cells adhere, grow and change morphology [19], the latter of which can arise from receptor stimulation as discussed in the introduction. C5aR activation was also profiled on the optical based EPIC^®^ system, where a readout in picometer correlates to “dynamic mass redistribution (DMR)” [58] changes at the sensor surface.

**Figure 4:**
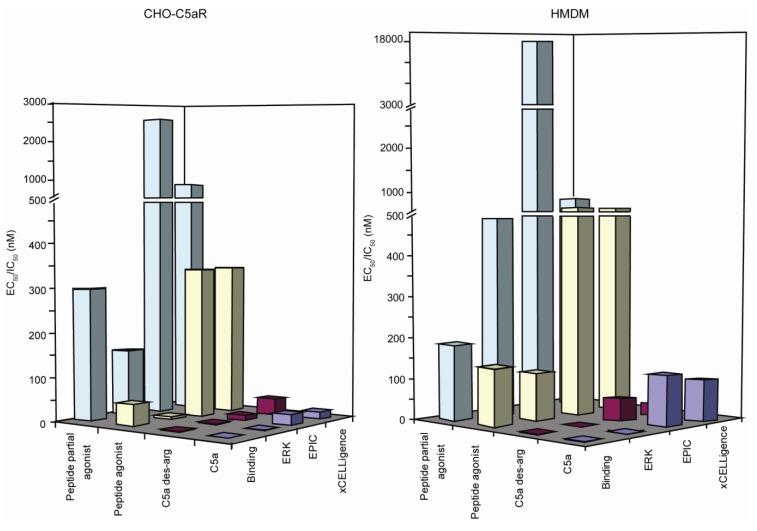
3D bar graph comparison of data across all platforms for the different ligands on CHO-C5aR cells and HMDM. Note break in x-axis scale at 500 and 3,000 nM.

It is understandable that being a relatively new technology that excludes the use of “labels”, in this field of science arouses questions regarding the exact nature of the signal. The signalling profiles generated on the two label-free platforms assessed in this study, are shown in Figure 2 and the summary of all the ligand potencies via different pathways, and the extent to which they are up- or down-regulated in our assays, is summarised in Figure 4. Since these technologies were first introduced, considerable effort has been invested into deciphering the nature of the signal and what it means in the biological context. Major cell events, such as cell growth and proliferation lead to large signals. However, GPCR receptor activation events lead to smaller changes in cell morphology and mass redistribution in the cell that can be monitored, as shown in this study, with the xCELLigence and EPIC^®^ systems, respectively. Orthogonal studies, along with pharmacological and chemical studies, have been used to propose that GPCRs that couple to different G proteins have unique DMR signalling profiles [59]. This was supported in a study by Schroder *et al.*, where they used the EPIC^®^ label-free system and found the signalling fingerprints to be in agreement with each of the major Gα protein classes proposed previously [60]. In our study, we observed a similar EPIC^®^ signalling profile for C5a, in both cell types (Figure 2), to that attributed to the Gαi protein in Schroder’s study. However, the signalling profile for C5a on the xCELLigence system differed not only to the EPIC^®^ profile, but between cell types. It is apparent from these profiles that the xCELLigence system yields more complex data and the profile for the same receptor varies significantly from cell type to cell type. The kinetics of the xCELLigence responses observed on the HMDM differ to the CHO-C5aR cells, specifically the sharp decline in the cell index after the peak is reached, a pattern not so apparent in the transfected cells. The decline in the HMDM signal observed after the peak on the EPIC^®^, is also slightly faster than that observed for the CHO-C5aR, however; it is not as prominent as that observed on the xCELLigence profile. As mentioned above, the C5L2 “scavenger” receptor is present in HMDM, and this may play a role in removing excess C5a, so that the signalling events are down regulated more rapidly and hence a more rapid decline is observed. Although we know that the signal generated on the label-free platforms examined in this study are due to the activation of C5aR (control cells showed no response), the exact nature (ERK, cAMP, calcium *etc*.) of the signal is unknown. It is beyond the scope of this study to decipher what causes the individual peaks shown in Figure 2. However, studies have been undertaken to understand what signalling components generate the DMR signal for receptors such as the EGF receptor. By utilising inhibitors for a range of known intracellular signalling components, Fang *et al.*, were able to show that the DMR profile for EGFR was attributable to Ras/MAPK signalling [58]. However, how these results translate across platforms is unknown. It would not be entirely surprising that signature profiles are consistent within a detection system, but differ between systems because the detection modes are different. For example, the Corning EPIC^®^ system used in this study, exhibits a sample penetration depth of ~150 nm [17], but the xCELLigence system has no such limit. However, the results summarised in Table 1 and Table 2 show that, despite this difference, the ligand potencies ascertained from both the label-free platforms were comparable. Indeed, for both of these platforms, the potencies were consistently lower than the potencies calculated using the secondary messenger reporter assays. This is not in agreement with the findings of Schroder *et al. *[60], where no significant difference between EPIC^®^ and secondary messenger assay potencies were reported. Interestingly, Dodgson *et al.* [61] found that the potencies of some compounds were similar between the Corning EPIC^®^ system and the FLIPR system, but with other compounds there was a 10-fold decrease in potency on the label-free platform compared to the label-based platform. The latter observation is similar to what has been found in this study. The shifts in potency on label-free platforms might be attributed to the signal representing an integrated response. All events ranging from cell stimulation to receptor recycling are measured and integrated, therefore a less potent response may be observed. By analogy, if one was to take individual potencies for all possible pathways activated upon receptor stimulation, including those that down-regulate the signalling response, and average them, the potency is more likely to reflect what is detected with label-free biosensors. In secondary messenger reporter assays, only downstream amplified secondary messengers are measured, which may present more potent responses [61] as no accountability is taken for signal regulation. It is not currently known whether the discrepancy between label-free and label-based assays is related to the type of GPCR assayed or characteristics of the ligand or both. 

Since the biosensors currently available for cell based plate assays are based on either impedance or optical detection, and we found no differences in ligand potencies between the two detection methods with the EPIC^®^ and xCELLigence, it is likely that other commercially available impedance and optical biosensors, yield comparable ligand potencies. However, significant differences in signalling profiles and the complexity of the data generated are possible, due to instrument configurations. Although the two label-free technologies sampled in this study yielded comparable ligand potencies their benefits and drawbacks are worth noting. One advantage of the xCELLigence system is its ability to capture fast early responses, which is not possible with the EPIC^®^ system. This is due to the inability of liquid handling to be performed directly onto the plate while it is reading, something the xCELLigence system can be configured to do. Data analysis, however, is more likely to be consistent between users for the EPIC^®^ system, as generally speaking the peak response is analysed. However, the xCELLigence software offers a number of analysis parameters, such as peak response, area under the curve, EC/IC_50_ over time *etc*. This can be useful when analysing samples where a peak response does not reflect the whole story.

## 4. Conclusions

Overall, this study has shown that: (i) both label-free technologies analysed in this study yield comparable results for activation of the human C5a receptor in endogenous and stably transfected cells; (ii) label-free cell responses for the C5aR display lower ligand potencies relative to secondary messenger reporter assays, but the rank order of potencies is similar; and (iii) native cells generally induce a lower response than stably transfected cells. The global cellular response, provided by label-free technologies, shows promise in the quantification of ligand action against GPCRs. We have demonstrated that it is possible to screen compounds in cells representative of a disease type, in this case a human inflammatory cell, rather than CHO cells transfected with a human receptor that involve different intracellular signal transduction pathways than for human cells. This advantage may help in the identification of “hits” that are less likely to fail at later stages of development for human conditions. Despite ongoing efforts to make assays ever more robust, “hits” identified in screening campaigns are failing to progress to the market due to unanticipated side effects and toxicities, some stemming from off-target binding, but others potentially the result of biased reporting of ligand function limited by one or a few secondary messenger reporter assays [62]. As label-free biosensors measure a global cellular response, their use may avoid some of the pitfalls associated with biased assessment of ligand function using secondary messenger assays. With high throughput formats available and high content information efficiently gained per label-free assay using limited sample amounts and little manipulation needed, one can foresee these technologies complementing routine GPCR label-based pathway specific assays in the near future.
